# Iterative learning control of neuronal firing based on FHN and HR models

**DOI:** 10.1371/journal.pone.0329380

**Published:** 2025-07-31

**Authors:** Chunhua Yuan, Xiaotong Wang, Xiangyu Li, Yueyang Zhao

**Affiliations:** 1 School of Automation and Electrical Engineering, Shenyang Ligong University, Shenyang, China; 2 Library, Shengjing Hospital of China Medical University, Shenyang, China; Buckinghamshire New University—High Wycombe Campus: Buckinghamshire New University, UNITED KINGDOM OF GREAT BRITAIN AND NORTHERN IRELAND

## Abstract

Neuronal firing patterns are fundamental to neural information processing and functional regulation, with abnormal firing closely linked to a range of neurological disorders. However, existing neuromodulation techniques largely rely on open-loop stimulation strategies, which lack adaptability and fail to provide precise control over neuronal dynamics. To address this limitation, this study introduces a novel iterative learning control (ILC) framework based on proportional-integral (PI) control for closed-loop modulation of neuronal firing patterns. The proposed method is developed and validated using two representative neuron models: the FitzHugh–Nagumo (FHN) and Hindmarsh–Rose (HR) models. A dynamical analysis of these models is conducted, followed by the design and implementation of a PI-based ILC strategy. Numerical simulations demonstrate that the proposed control method significantly outperforms conventional PI control, achieving lower tracking errors, enhanced control accuracy, and improved system stability. Additionally, the ILC approach exhibits strong adaptability to different neuronal dynamics, highlighting its potential for precise and robust regulation in complex neural systems. These findings offer a theoretical basis for advancing closed-loop neuromodulation technologies, with promising implications for applications in neurorehabilitation and the treatment of neurological disorders.

## 1. Introduction

The nervous system forms the core framework for information processing in living organisms. Its operation relies heavily on the precise coordination of neuronal firing activities, which underpin neural coding strategies and the transmission of signals throughout the brain. These dynamics are essential for fundamental functions such as perception, movement control, and higher-order cognition. Over the decades, researchers have developed a range of mathematical models to describe neuronal electrical activity, aiming to capture the essential characteristics of different types of neurons. Among these, several simplified and representative models—such as those inspired by early experimental findings—have been widely adopted to explore neuronal behavior and inform theoretical studies [1].

With the deepening integration of control theory and neuroscience, recent research has increasingly adopted ILC methodologies for regulating neuronal firing behavior. Zhang, Dai, and colleagues introduced an entropy-based ILC approach designed for the estimation and control of stochastic nonlinear neural systems, effectively harnessing historical interactions among neuronal membrane potentials to enable dynamic identification and optimized regulation [2]. Tang et al. further extended this framework to address the identification of coupling characteristics in membrane potentials, employing entropy metrics to uncover latent information transmission mechanisms within the coupling structures [3]. Additionally, Zhang and Sepulveda applied this methodology to model inter-axonal interactions, proposing an ILC-based strategy for axonal connectivity identification [4]. Moreover, Zhang and Zhou provided a comprehensive review of recent advances in the control of non-Gaussian stochastic systems, emphasizing the theoretical significance and practical potential of entropy-informed ILC frameworks in bio-information systems [5].

In the realm of neuromodulation and neural system modeling, achieving high-precision control within complex, nonlinear neural dynamical systems using advanced learning algorithms and optimization strategies has emerged as a critical and challenging research frontier [6–8]. Conventional control approaches, such as PID or PI controllers, primarily focus on asymptotic trajectory tracking. However, their performance and robustness are often compromised when dealing with neural systems characterized by strong nonlinearity, high uncertainty, and non-Gaussian disturbances.

Although clinical technologies such as deep brain stimulation (DBS) have been widely employed in the treatment of movement disorders, Putzke et al. emphasized that the optimization of stimulation parameters is critical for improving therapeutic efficacy [9]. However, most current neuromodulation techniques still rely on open-loop stimulation schemes and lack adaptive capabilities tailored to the individual dynamical characteristics of neurons, thereby limiting their practicality in high-precision control scenarios. Traditional linear control methods have also shown evident limitations when applied to complex neural systems, often failing to effectively address the strong nonlinear behaviors and structurally coupled dynamics inherent in neuronal networks. Consequently, developing control mechanisms for neural systems that are both self-adaptive and robust has become an urgent and essential research direction.

Inspired by the aforementioned studies, this work proposes a novel ILC)strategy based on proportional-integral PI control, specifically designed for two representative neuron models—the FHN model and the HR model. This approach integrates conventional PI feedback with a historical error correction mechanism, enabling adaptive adjustment to diverse neuronal dynamics and facilitating progressive convergence toward a desired spiking trajectory. Through numerical simulations, the proposed method is shown to significantly improve tracking accuracy, enhance system stability, and maintain effectiveness under various electrical stimulation conditions. A systematic comparison with traditional control techniques further validates the superior performance of the proposed scheme.

This study not only provides a methodological framework for understanding the regulation of complex neuronal electrical activity but also offers theoretical guidance for the design of closed-loop control strategies in future neuromodulation devices and brain–machine interface systems.

## 2. Model and methods

### 2.1. HR neuron model

Hindmarsh and Rose introduced a mathematical model to simulate the electrical activity of neurons, particularly inspired by experimental observations of snail neurons. The HR model has since become a widely adopted tool for exploring complex neuronal firing patterns and dynamical behaviors. It is especially valuable in studies of intrinsic neuronal excitability and synaptic interactions due to its ability to reproduce a variety of spiking and bursting phenomena observed in real neurons [10]. The HR neuron model can be expressed as the following three-dimensional system of differential equations:


$$\{\;\;\;\begin{array}{c} {}*20c\frac{dx}{dt}=y-ax^{3}+bx^{2}-z+I\mathrm{\backslash vspace}2mm\\ \mathrm{\backslash vspace}2mm\frac{dy}{dt}=c-dx^{2}-y\\ \;\;\;\;\;\;\;\frac{dz}{dt}=r(s(x-x_{0})-z) \end{array}$$ (1)

In this model, x represents the membrane potential of the neuron, while y and z are auxiliary variables that describe the activation and recovery processes, respectively. The parameters a,b,c,d,r,s, and I are system constants or input terms. Specifically, I represents the input current, which determines the excitability of the neuron.

### 2.2. FHN neuron model

The FitzHugh–Nagumo (FHN) model is one of the most fundamental models in computational neuroscience for describing neuronal excitability. It originated from a simplified version of the Hodgkin–Huxley model, capturing essential dynamical features of neuronal behavior while significantly reducing mathematical complexity. Later, Nagumo and colleagues implemented an electronic analog of the model using a tunnel diode, further validating its practical relevance [11]. It plays a crucial role in biophysics and computational neuroscience [12–14]. The FHN neuron model can be expressed as the following two-dimensional system of differential equations:


$$\{\;\;\;\begin{array}{c} {}*20c\frac{dv}{dt}=v-\frac{v^{3}}{3}-w+I_{ext}\\ \frac{dw}{dt}=\varepsilon (v+a-bw) \end{array}$$ (2)

In this model, v represents the membrane potential variable, while w is the recovery variable. The term $I_{ext}$ denotes the external stimulus current. The parameter ε is a small constant that controls the time scale of the recovery variable. The parameters a and b are model coefficients that regulate the dynamical behavior of the neuron.

### 2.3. Iterative control algorithm based on PI control

Controlling the generation of desired or normal neuronal firing patterns is one of the key areas in closed-loop electrophysiological research. The core idea of Iterative Learning Control is to achieve better control performance through repeated control actions. For any task with repetitive characteristics, opportunities to improve task execution performance can be identified based on observations from the previous execution, which can then be used to enhance the next execution [15]. In this study, controlling the neuronal model is treated as a control task, with the goal of tracking a given reference trajectory. This task is accomplished by gradually adjusting the control input to minimize tracking errors. Based on the mathematical descriptions of the FHN and HR neuron models, we consider the following continuous-time, multivariable nonlinear dynamical system:


$$\{\;\;\;\begin{array}{c} {}*20c\dot{x}(t)=\text{f}\left(t,x(t),u(t)\right)\\ y(t)=g(t,x(t))+D(t)u(t) \end{array}$$ (3)

In this system, $x\in R^{n}$ represents the state vector, $y\in R^{m}$ is the output vector, and $u\in R^{r}$ is the input vector. The nonlinear functions f and g have unknown but bounded structures and parameters. D is a real matrix of appropriate dimensions.

The problem of Iterative Learning Control can be stated as follows: Given a reference desired trajectory $y_{d}(t)$ over the time interval [0,T] for the system described by equation (3), the objective is to find an iterative control law such that the system generates a perfect tracking trajectory $y_{d}(t)$.

Assuming the number of iterations is k, the dynamics of the k−th iteration are given by:


$$\{\;\;\;\begin{array}{c} {}*20c{\dot{x}}_{k}(t)=f(t,x_{k}(t),u_{k}\left(t)\right)\\ y_{k}(t)=g(t,x_{k}(t))+D(t)u_{k}(t) \end{array}$$ (4)

The tracking error at the k−th iteration is given by:


$$e_{k}(t)=y_{d}(t)-y_{k}(t)$$ (5)

In general, the closed-loop PI-type Iterative Learning Control law is a fundamental iterative learning algorithm. The input at the k−th iteration is the sum of the control input at the (k+1)−th iteration and a PI correction term based on the (k+1)−th output error, given by:


$$u_{k+1}(t)=u_{k}(t)+Kpe_{k+1}(t)+Ki\int_{0}^{t}e_{k+1}(s)ds$$ (6)

**Fig 1:**
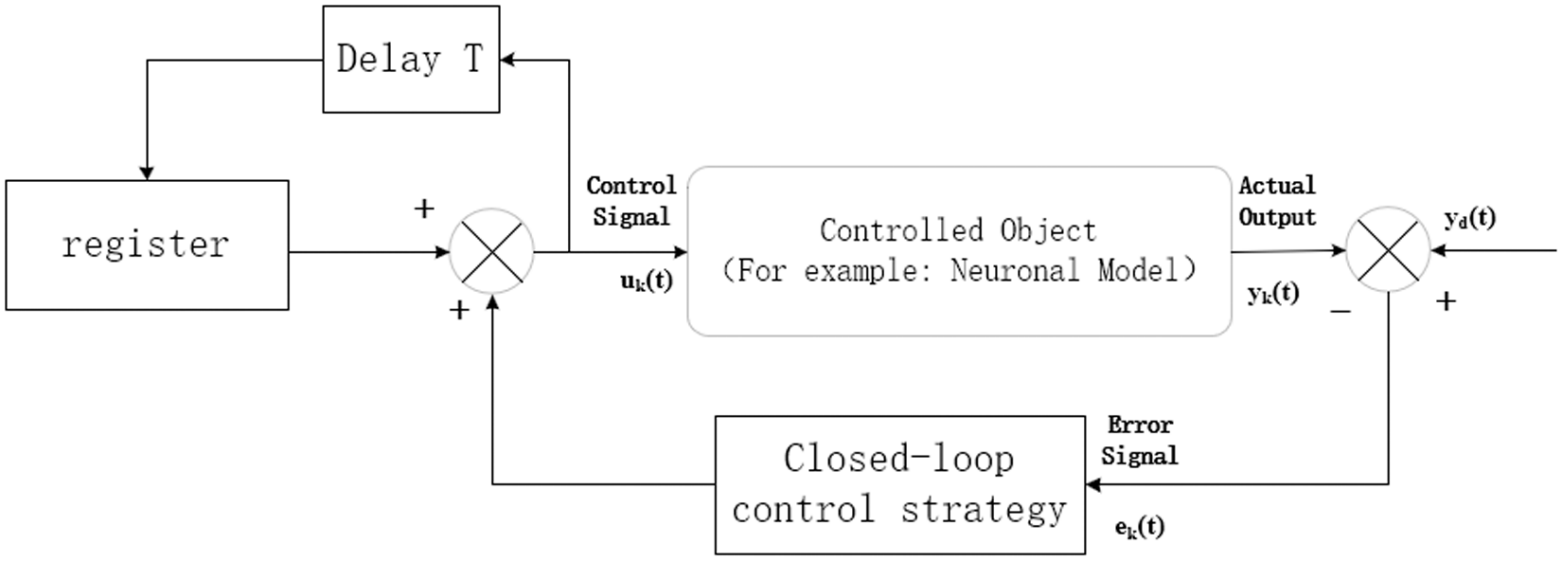
Basic structure of closed-loop Iterative Learning Control.

In the above equation, *Kp* and *Ki* are the PI gain matrices. The basic structure of the closed-loop PI-type Iterative Learning Control is shown in Fig 1.

## 3. Results

The core function of the nervous system lies in the efficient processing of information, and the firing patterns of neurons play a crucial role in this process. Studies have shown that neuronal firing behavior is influenced by both intrinsic dynamical characteristics and externally applied stimuli, resulting in various complex firing patterns, such as single-pulse firing, periodic spike firing, and burst firing [16,17]. These firing patterns not only affect the encoding and transmission of neural information but also directly determine the functional state of neural circuits.

### 3.1. Control of HR neuronal model firing dynamics

In this section, numerical simulations of the HR neuron model are conducted using both PI control and closed-loop ILC. The periodic burst firing pattern is used as the desired firing trajectory. The HR neuron model is subjected to direct current stimulation under ideal, noise-free conditions to observe the effects of both control methods I=2.75μA,sinusoidal current stimulation: I=2.75+sin(0.02πt)μA Two types of electrical stimuli are applied to observe the effects of both control methods. Under Iterative Learning Control, a total of 20 iterations are performed, with each iteration having a simulation step size of 0.01 ms, resulting in a total simulation time of 1600 ms. The parameters for Iterative Learning Control are chosen as Kp=10 Ki=0.05 The parameters for PI control are set as Kp=10, Ki=0.05, The total simulation time is 1600 × 20 = 32000ms, with the same simulation step size as used in Iterative Learning Control.

**Fig 2:**
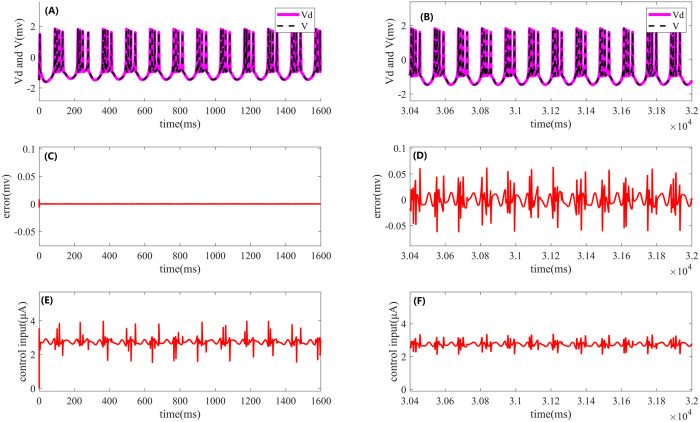
Effects of Iterative Learning Control and PI control on periodic cluster firing of the HR neuron model under direct current stimulation. The left side shows the results of ILC, while the right side presents the corresponding effects of PI control. (A) and **(B)**: Desired output (pink solid line) and actual output (black dashed line) of the HR neuron model under ILC and PI control. (C) and **(D)**: Comparison of the error between the desired and actual outputs during the periodic cluster firing process under both control methods. (E) and **(F)**: Control signals of ILC and PI control.

First, direct current stimulation is applied under noise-free conditions. For comparison, the simulation results are summarized in a single fig, as shown in Fig 2. The left side of Fig 2 presents the results of Iterative Learning Control, while the right side corresponds to the effects of PI control. In Fig 2A and 2B, the pink solid line represents the desired firing waveform under open-loop stimulation in a noise-free environment, while the black dashed line indicates the actual output of the HR model under closed-loop PI or ILC control. Fig 2C shows the error between the desired and actual outputs during the 20th iteration of ILC, while Fig 2D depicts the control error of PI control over the final 1600 ms. A comparison of Fig 2C and 2D reveals that the error under ILC is significantly smaller, nearly forming a straight line, indicating superior control performance. Furthermore, Fig 2E and 2F illustrate the control signals at the final iteration of ILC and the control signal of PI control over the last 1600 ms, respectively.

**Fig 3:**
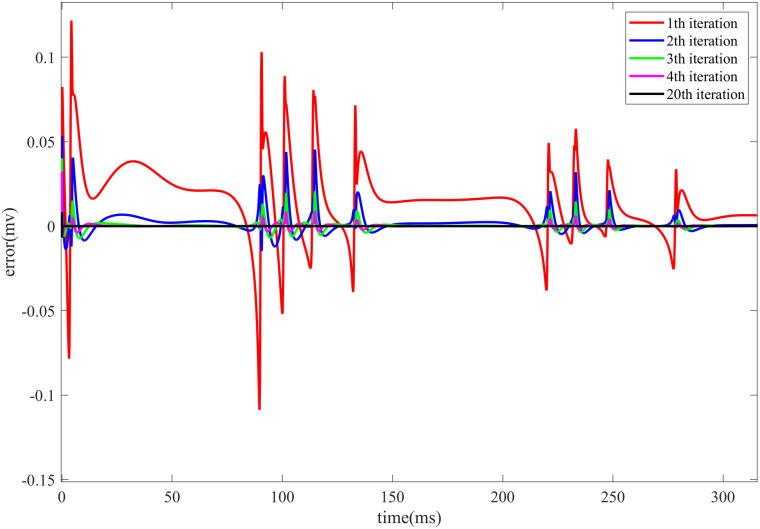
Evolution of the control error in the HR neuron model during the first 300 ms of Iterative Learning Control under DC) stimulation. The fig shows the change in control error with respect to the iteration number, including the error evolution during the first four iterations and the final iteration.

Fig 3 illustrates the evolution of the control error in the HR neuron model during Iterative Learning Control under direct current stimulation. The fig presents the trend of the error over time during the first four iterations, as well as the error at the final iteration.

**Fig 4:**
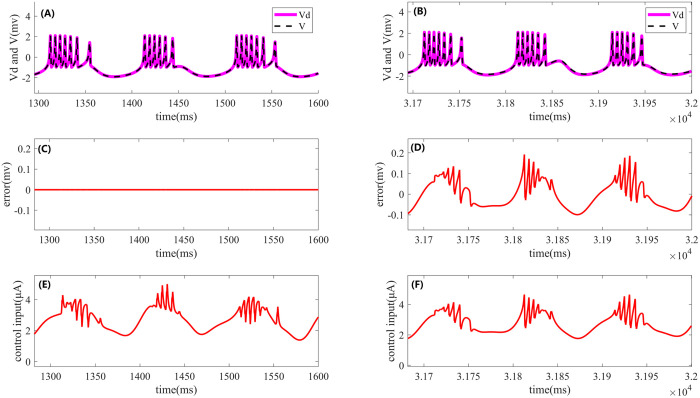
Effects of Iterative Learning Control and PI control on periodic cluster firing of the HR neuron model under sinusoidal current stimulation. The left side shows the results of ILC, while the right side displays the corresponding effects of PI control. (A) and **(B)**: Desired output (pink solid line) and actual output (black dashed line) of the HR neuron model under ILC and PI control. (C) and **(D)**: Comparison of the error between the desired and actual outputs during the periodic cluster firing process under both control methods. (E) and **(F)**: Control signals of ILC and PI control.

Let me know if you would like to expand or rephrase any other part of the text. I=2.75+sin(0.02πt)μA In ideal noise-free conditions, the control effects of the HR neuron model under sinusoidal current stimulation were observed. As shown in Fig 4, the left side corresponds to Iterative Learning Control, while the right side represents PI control. Specifically, Fig 4A and 4B display the neuron firing waveforms under both control methods. The pink solid line represents the desired firing trajectory in the noise-free environment, while the black dashed line corresponds to the output of the closed-loop PI or Iterative Learning Control. Fig 4C shows the error at the 20th iteration of Iterative Learning Control during the final 300 ms, and Fig 4D presents the error curve for PI control during the final 300 ms. It is evident that the error for Iterative Learning Control is smaller and almost forms a straight line, demonstrating its superior performance. Additionally, Fig 4E and 4F show the control signals for Iterative Learning Control during the final iteration and for PI control during the last 300 ms.

**Fig 5:**
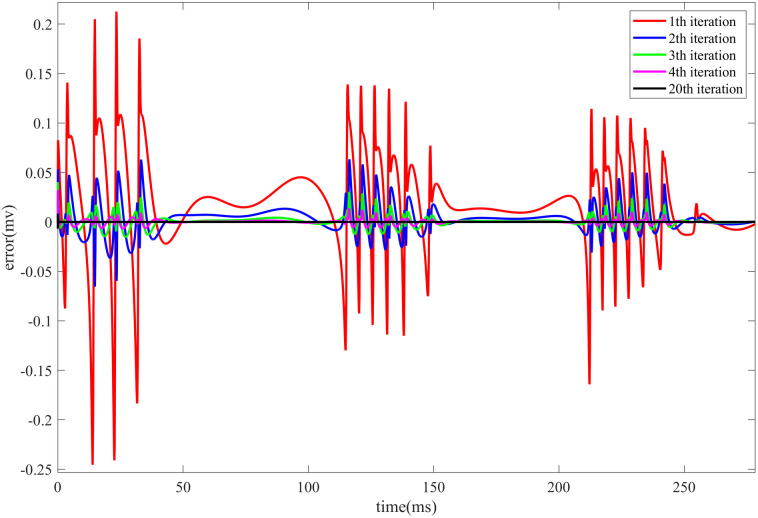
Control error evolution during the Iterative Learning Control process of the HR neuron model under sinusoidal current stimulation over the first 250 ms. The fig includes the error trends for the first four iterations as well as the final iteration.

Fig 5: Evolution of the control error during the Iterative Learning Control process of the HR neuron model under sinusoidal current stimulation. The fig includes the error trends for the first four iterations as well as the final iteration.

In this section, numerical simulations of the HR neuron model were conducted using closed-loop PI-type Iterative Learning Control and classical PI control. The simulation results demonstrate that, under Iterative Learning Control, the HR neuron’s actual output quickly tracks the desired trajectory, with minimal fluctuation in the control input, thereby reducing potential damage to the neuron. In contrast, the tracking performance of the classical PI control is weaker. A further comparison reveals that, under Iterative Learning Control, the error between the actual and desired outputs is significantly smaller, indicating superior control performance and effectiveness in tracking the desired firing pattern.

### 3.2. Control of FHN neuronal model firing dynamics

This section presents numerical simulations of the FHN neuron model using both PI control and closed-loop Iterative Learning Control, with periodic cluster firing set as the desired firing trajectory. To evaluate the effectiveness of different control strategies, both DC and sinusoidal current stimulations are applied in an ideal noise-free environment, in order to observe the responses under each control method.

**Fig 6:**
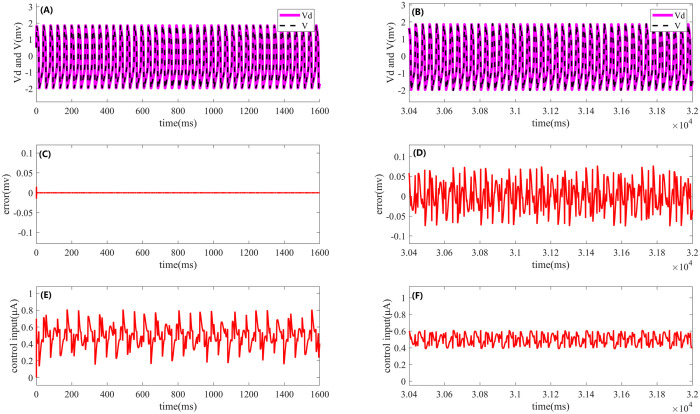
Performance of iterative learning control and PI control in FHN neuron model periodic cluster discharge simulation. The left side shows the results of iterative learning control, while the right side shows the results of PI control. (A) and **(B)**: Desired output (pink solid line) and actual output (black dashed line) under both control strategies. (C) and **(D)**: Comparison of the errors between the desired and actual outputs for the two control methods. (E) and **(F)**: Control signals for iterative learning control and PI control, respectively.

Apply DC stimulation: I=0.5μA and Sinusoidal current stimulation $I=\frac{1}{2}+\sin(0.004\pi t)\mu A$ Two types 1of electrical stimulation were applied, and under iterative learning control, a total of 20 iterations were performed, with a simulation step size of 0.01 ms and a total simulation time of 1600 ms per iteration The parameters for iterative learning control were set as follows: Kp=1.5, Ki=0.15,The parameters for PI control were set as follows: Kp=1.5, Ki=0.15,The simulation time was set to 1600 × 20 = 32000 ms, with the step size consistent with that of the iterative learning control. Under noise-free conditions, a DC stimulus was applied to the FHN neuron model. For comparison of different control strategies, the simulation results were consolidated into a single fig, as shown in Fig 6. The left side of Fig 6 presents the results of the iterative learning control, while the right side corresponds to the performance of PI control. From the fig, it is evident that the tracking error under iterative learning control is smaller, and the control signal is smoother. Compared to PI control, the overall control performance is superior.

In the case of DC stimulation, as shown in Fig 6A and 6B, the pink solid line represents the ideal periodic cluster discharge waveform under open-loop stimulation, while the black dashed line corresponds to the actual output of the FHN model under closed-loop PI-type iterative learning control or classic PI control. Fig 6C presents the error between the desired and actual outputs during the final iteration of the iterative learning control, while Fig 6D shows the control error under PI control. By comparing Fig 6C and 6D, it can be observed that the error in iterative learning control approaches zero, whereas the error in PI control is relatively larger, indicating the superior control precision of the former. Additionally, Fig 6E and 6F display the control signals for both control strategies, showing that the signal in iterative learning control is smoother.

**Fig 7:**
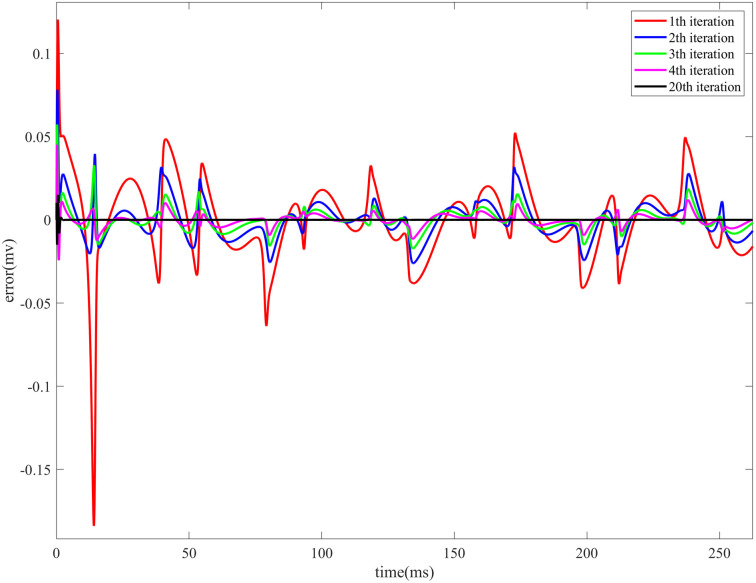
Evolution of the error in the FHN neuron model under direct current stimulation during the iterative learning control process over the first 250ms. The fig includes the error behavior for the first four iterations and the final iteration.

Fig 7: Evolution of the error in the iterative learning control process of the FHN neuron model under direct current stimulation. This fig shows the error trend over time, including the changes in error during the first four iterations and the final iteration.

**Fig 8:**
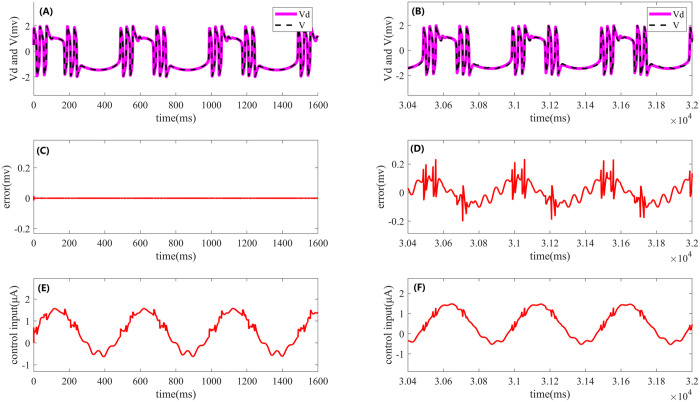
Comparison of iterative learning control and PI control in the periodic clustered firing simulation of the FHN neuron model. The left side shows the iterative learning control, while the right side presents the PI control. (A) and (B) display the expected output (pink solid line) and actual output (black dashed line) of the FHN model under both control strategies. (C) and (D) provide error analysis during the periodic clustered firing control process for both control strategies. (E) and (F) present the control signals at the final iteration for iterative learning control and within the last 1600 ms for PI control, respectively.

Under sinusoidal current stimulation $I=\frac{1}{2}+\sin(0.004\pi tmuA$ a direct current stimulus was applied to the FHN neuron model under ideal noise-free conditions, as shown in Fig 8. The left side of Fig 8 presents the simulation results for iterative learning control, while the right side illustrates the performance of the PI control. Specifically, Fig 8A and 8B show the neuronal firing waveforms under both control strategies. The pink solid line represents the ideal open-loop stimulation waveform under noise-free conditions, while the black dashed line indicates the actual output of the FHN model under closed-loop PI iterative learning control or conventional PI control. Fig 8C depicts the error at the 20th iteration of iterative learning control, whereas Fig 8D shows the error within the final 1600 ms of conventional PI control. A comparison reveals that the error of the iterative learning control is nearly linear and significantly smaller than that of the PI control, further confirming its superior control performance. Additionally, Fig 8E and 8F present the control signals for the two control strategies.

**Fig 9:**
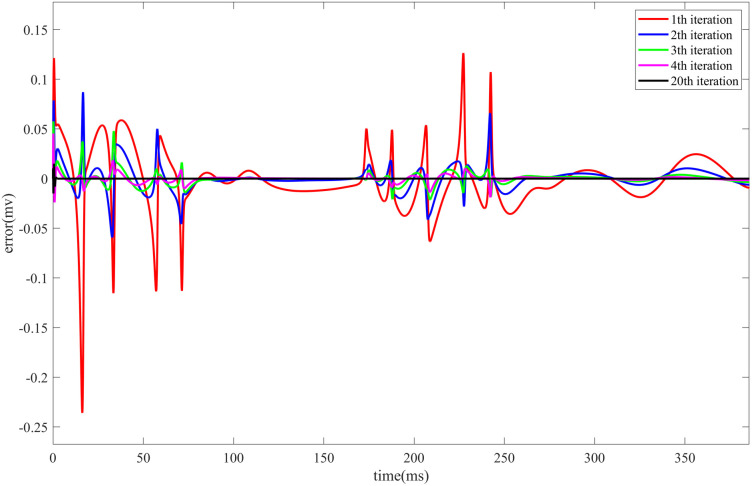
The evolution of the control error over the course of the iterative learning control process in the FHN neuron model under sinusoidal current stimulation during the first 350 ms, including the error trends for the first four iterations and the final iteration.

Fig 9 shows the variation in control error of the FHN neuron model under sinusoidal current stimulation, detailing the temporal evolution of the error during the first four iterations and the final iteration.

## 4. Discussion

The comparison between the performance of the novel iterative learning control and traditional PI control on the HR and FHN neuron models through numerical simulations leads to the following key conclusions:

(1) Control Accuracy and Error Convergence

The simulation results indicate that the novel ILC significantly reduces the tracking error of the neuronal firing patterns. Both in the HR and FHN models, ILC demonstrates superior tracking performance compared to PI control. As the iteration progresses, the ILC error quickly converges and stabilizes, demonstrating its effectiveness in complex nonlinear systems. In contrast, the PI control exhibits larger errors, slower convergence, and, in some cases, difficulty in achieving complete convergence.

(2) Adaptability to Different Stimulation Modes

The simulations were conducted using both direct current and sinusoidal current stimulation. The results show that the novel ILC method adapts well to different stimulation modes, maintaining stable control performance under both conditions. This adaptability indicates that the novel ILC method is highly versatile and can be extended to more complex neural regulation tasks. In contrast, the control performance of PI control varies significantly across different stimulation modes, indicating poorer robustness.

(3) Idealized Simulation Conditions

All simulations were conducted under noise-free and idealized conditions. In real biological systems, neuronal activity is subject to various types of disturbances, such as thermal noise, synaptic variability, and parameter uncertainties. The performance of the proposed ILC method under such non-ideal, real-world scenarios remains to be evaluated.

## 5. Conclusion

This study investigates a novel iterative control method based on PI control for regulating neuronal firing patterns, with simulations conducted using the FHN and HR neuron models. The results demonstrate that the new control method effectively reduces tracking errors, enabling the neuronal firing trajectory to quickly converge to the target pattern while maintaining high stability under different stimulation conditions. Compared to traditional PI control, the novel method not only improves tracking accuracy but also minimizes potential interference with the nervous system.

Moreover, this method adapts to the dynamic characteristics of different neuron models, exhibiting superior control performance under both DC and sinusoidal stimulation for both HR and FHN models. Overall, the novel iterative control method overcomes the limitations of traditional linear control approaches in the complex nonlinear systems of neurons, providing a more precise and stable solution for neural regulation.

The findings of this research not only offer new theoretical support for interventions in neurological disorders and the development of neurorehabilitation technologies but also have broad applications in fields such as brain-like computing and neural engineering. Future research could further integrate machine learning and optimization algorithms to enhance the intelligence of the iterative control strategy and explore its applicability in more complex neural systems. In conclusion, this study confirms the effectiveness of the novel iterative learning control in neuronal firing pattern regulation, highlighting its significant research value in neural regulation and intelligent control. Future studies can explore its application in more complex neural system models and integrate machine learning or optimization algorithms to improve the adaptability and intelligence of the control strategy.

## Supporting information

S1 File Supporting Information. This ZIP file contains all supplementary materials, including the raw data supporting the figures and tables presented in the article.
(ZIP)

## References

[1] Hodgkin AL, Huxley AF. Currents carried by sodium and potassium ions through the membrane of the giant axon of Loligo. J Physiol. 1952;116(4):449–72. doi: 10.1113/jphysiol.1952.sp004717 14946713 PMC1392213

[2] Zhang Q, Dai X. Entropy-based iterative learning estimation for stochastic non-linear systems and its application to neural membrane potential interaction. 2019 1st International Conference on Industrial Artificial Intelligence (IAI). IEEE; 2019.

[3] Tang X, Zhang Q, Dai X, Zou Y. Neural membrane mutual coupling characterisation using entropy-based iterative learning identification. IEEE Access. 2020;8:205231–43.

[4] Zhang Q, Sepulveda F. Entropy-based axon-to-axon mutual interaction characterization via iterative learning identification. In: European Medical and Biological Engineering Conference. Springer; 2017. p. 691–4.

[5] Zhang Q, Zhou Y. Recent advances in non-Gaussian stochastic systems control theory and its applications. Int J Network Dynamic Intell. 2022:111–9.

[6] Mou J, Cao H, Zhou N, Cao Y. An fhn-hr neuron network coupled with a novel locally active memristor and its dsp implementation. IEEE Trans Cybernetics. 2024. 10.1109/TCYB.2024.3471644 39383075

[7] Baysal V, Solmaz R, Ma J. Investigation of chaotic resonance in Type-I and Type-II Morris-Lecar neurons. Appl Math Comput. 2023;448:127940.

[8] Ghanbarpour G, Hoque A, Assaad M, Ghanbarpour M. New model for Wilson and Morris–Lecar neuron models: validation and digital implementation on FPGA. IEEE Access. 2024.

[9] Putzke JD, Wharen RE Jr, Wszolek ZK, Turk MF, Strongosky AJ, Uitti RJ. Thalamic deep brain stimulation for tremor-predominant Parkinson’s disease. Parkinsonism Relat Disord. 2003;10(2):81–8. doi: 10.1016/j.parkreldis.2003.09.002 14643997

[10] Hindmarsh JL, Rose RM. A model of the nerve impulse using two first-order differential equations. Nature. 1982;296(5853):162–4. doi: 10.1038/296162a0 7063018

[11] FitzHugh R. Mathematical models of threshold phenomena in the nerve membrane. Bulletin Math Biophys. 1955;17:257–78.

[12] Xu Q, Chen X, Chen B, Wu H, Li Z, Bao H. Dynamical analysis of an improved FitzHugh-Nagumo neuron model with multiplier-free implementation. Nonlinear Dynamics. 2023;111(9):8737–49.

[13] Wang Z, Chen M, Xi X, Tian H, Yang R. Multi-chimera states in a higher order network of FitzHugh–Nagumo oscillators. European Phys J Special Topics. 2024;233(4):779–86.

[14] Yasin MW, Iqbal MS, Ahmed N, Akgül A, Raza A, Rafiq M, et al. Numerical scheme and stability analysis of stochastic Fitzhugh–Nagumo model. Results Phys. 2022;32:105023. doi: 10.1016/j.rinp.2021.105023

[15] Tan Y, Dai H-H, Freeman C, editors. A dual iterative learning control loops for cascade systems. 2012 24th Chinese Control and Decision Conference (CCDC). IEEE; 2012.

[16] Qiao S, An XL. Dynamic response of the e-HR neuron model under electromagnetic induction. Pramana. 2021;95(2):72.

[17] Zheng Y, Hu X. Concurrent Prediction of Finger Forces Based on Source Separation and Classification of Neuron Discharge Information. Int J Neural Syst. 2021;31(6):2150010. doi: 10.1142/S0129065721500106 33541251

